# Preparation and Characterization of an Optimized Meniscal Extracellular Matrix Scaffold for Meniscus Transplantation

**DOI:** 10.3389/fbioe.2020.00779

**Published:** 2020-07-09

**Authors:** Yong He, Yunbin Chen, Xinyu Wan, Chenchen Zhao, Pengcheng Qiu, Xianfeng Lin, Jianfeng Zhang, Yue Huang

**Affiliations:** ^1^Department of Orthopaedic Surgery, Sir Run Run Shaw Hospital, Medical College of Zhejiang University, Hangzhou, China; ^2^Key Laboratory of Musculoskeletal System Degeneration and Regeneration Translational Research of Zhejiang Province, Hangzhou, China; ^3^Department of Neurology, The First Affiliated Hospital of Wenzhou Medical College, Wenzhou, China; ^4^First Clinical College, Wenzhou Medical University, Wenzhou, China

**Keywords:** meniscus, extracellular matrix, biomechanics, region-specific recellularization, decellularization

## Abstract

Many studies have sought to construct a substitute to partially replace irreparably damaged meniscus. Only the meniscus allograft has been used in clinical practice as a useful substitute, and there are concerns about its longevity and inherent limitations, including availability of donor tissue and possibility of disease transmission. To overcome these limitations, we developed an acellular xenograft from whole porcine meniscus. Samples were treated with 2% Triton X-100 for 10 days and 2% sodium dodecyl sulfate for 6 days. The DNA content of extracellular matrix (ECM) scaffolds was significantly decreased compared with that of normal porcine menisci (*p* < 0.001). Histological analysis confirmed the maintenance of ECM integrity and anisotropic architecture in the absence of nuclei. Biochemical and biomechanical assays of the scaffolds indicated the preservation of collagen (*p* = 0.806), glycosaminoglycan (*p* = 0.188), and biomechanical properties (elastic modulus and transition stress). The scaffolds possessed good biocompatibility and supported bone marrow mesenchymal stem cells (BMSCs) proliferation for 2 weeks *in vitro*, with excellent region-specific recellularization *in vivo*. The novel scaffold has potential value for application in recellularization and transplantation strategies.

## Introduction

The meniscus of the knee is fibrocartilaginous in nature, with an organized arrangement of collagenous fibers. The main functions of human menisci are load transmission, stress distribution, stability, and lubrication of the joint, which collectively prevent cartilage damage (Stapleton et al., 2011). Damage or degeneration of the meniscus is usually followed by loss of these functions and development of knee arthritis (Ding et al., 2007). Meniscus lesion repair has always been a great challenge in orthopaedic surgery because of its limited vascularization and capacity for self-regeneration. When damage involves the non-vascularized areas, meniscus preservation or restoration is difficult to achieve. Development of novel therapeutic methods for meniscus repair is both timely and necessary (Maier et al., 2007).

Many materials have been used to repair meniscus defects after (partial) meniscectomy. These include natural polymers (Stone et al., 1992), synthetic polymers (Klompmaker et al., 1996), allogenic meniscus (van Arkel and de Boer, 2002; Verdonk, 2002), and autologous tissue (Kohn et al., 1997; Bruns et al., 1998). A goal of polymer research in this regard is to mimic the organization and interactions among the major tissue constituents of natural meniscus. However, long-term studies have indicated the limited success of this approach. Meniscal allograft transplantation has been applied in patients worldwide, since the first successful operation in 1984 (Milachowski et al., 1989). Meniscal allografts produced good to excellent results regarding pain and function (Cameron and Saha, 1997; Rath et al., 2001; Noyes et al., 2005; Cole et al., 2006; Ha et al., 2011). Nevertheless, concerns over longevity and inherent limitations of the allografts, including limited graft supply and possible disease transmission (Noyes and Barber-Westin, 2010), mean they have limited wide application. Furthermore, while they are safe and available, properties of fat pad, quadriceps, and Achilles tendon autografts have been inferior to the allograft (Messner et al., 1999).

Acellular xenogenic meniscal tissue has similar anatomy and matrices to the human meniscus and is easy to access, and thus, it might be a promising alternative for transplantation. Many different protocols have been described for preparing meniscus extracellular matrix (ECM) scaffolds (Maier et al., 2007; Stapleton et al., 2008; Sandmann et al., 2009; Stabile et al., 2010; Azhim et al., 2013; Chen et al., 2015; Wu et al., 2015). The dense fibrous structure of the meniscus makes it difficult to undergo decellularization while retaining the maximum amount of bioactive molecules and biomechanical potential (Stapleton et al., 2008; Ionescu et al., 2011). Maier et al. (2007) reported the complete cell removal in whole ovine meniscus using an enzymatic solution. However, there was appreciable disruption and digestion of the ECM. In 2008, Sandmann et al. (2009) incubated human meniscus tissue in 2% sodium dodecyl sulfate (SDS) for 2 weeks and achieved cell removal. However, such a long SDS treatment resulted in greater cytotoxicity and ECM injury than treatment with Triton X-100 or tri-(n-butyl) phosphate (Cartmell and Dunn, 2004; Gilbert et al., 2006). Acellular ECM meniscal hydrogels as well as ECM meniscal fragments based on dissection and sonication have also been explored (Azhim et al., 2013; Wu et al., 2015), but the lower biomechanical potential of these materials is an important limitation.

In this study, we optimized the previous methods and developed a novel protocol for the decellularization of whole porcine meniscus, which is very similar in size to human menisci. The scaffolds preserved the bioactivity, biomechanics, and cytocompatibility features of the tissue, and excellent region-specific recellularization was observed *in vivo*.

## Materials and Methods

### Preparation of Acellular Meniscus Scaffolds

**FIGURE 1:**
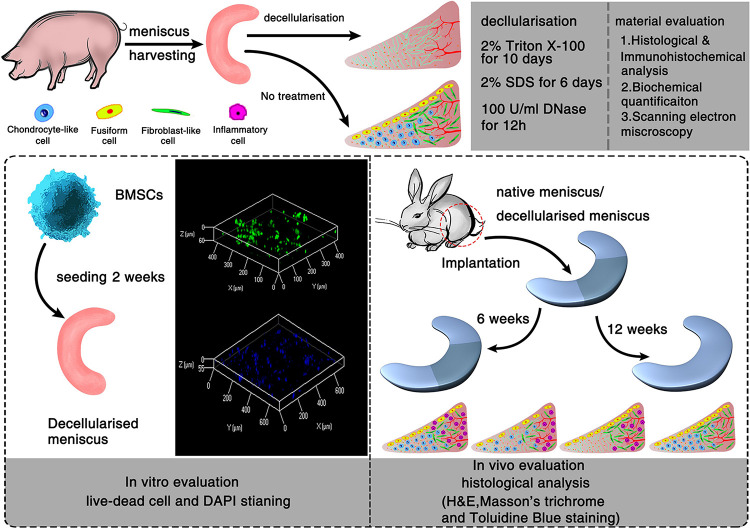
Schematic illustration of the decellularization process of porcine menisci and regeneration of decellularized meniscus. *In vitro* and *in vivo* evaluation of meniscus scaffolds with/without decellularization.

Six male pigs (6-month-old, Large White) were obtained from the Animal Experimental Center of Zhejiang University. All animal experiments were approved by the Animal Experimental Ethics Committee of Zhejiang University, and the animals were treated according to approved experimental protocols. Both medial and lateral menisci were harvested from pigs and divided into two groups, the normal group (*n* = 6) and decellularized group (*n* = 6). The menisci were then washed in phosphate buffered saline (PBS) to remove excess blood. Samples in the control group were immediately stored at 4°C for biochemical analysis, in 4% formaldehyde for histological analysis, and in 4% glutaraldehyde for scanning electron microscopy (SEM). Before determining the optimal decellularization protocol, Menisci were suspended in 2% Triton X-100 (Sigma-Aldrich, St. Louis, MO, United States) for 10 days and 2% SDS (Sigma-Aldrich) for 6 days, followed by deionized water (48 h) and PBS treatment (24 h) to wash out all remnants of detergent. To minimize the DNA content in the ECM, 100 U/ml DNAase (Sigma-Aldrich) in PBS was applied (Figure 1). All steps were performed at room temperature with agitation (100 rpm). Samples were stored in PBS at 4°C within 2 weeks of processing.

### Histological Analysis

Normal menisci (NM) and decellularized menisci (DM) were fixed for 24 h in a 10% neutral buffered formalin solution at room temperature for 24 h, then dehydrated automatically by gradient concentrations of ethanol (50, 70, 80, 90, and 100%, for 5 min each), and treated by xylol for 20 min before embedding in paraffin wax. Sections 5 mm in thickness were used for histological staining. Standard hematoxylin and eosin (H&E) staining was used to evaluate tissue microstructure and remaining cells. Alcian blue staining (1% w/v, pH = 2.5) was used for the qualitative analysis of glycosaminoglycan (GAG) content. Cell nuclei were counterstained with 1% neutral red stain. Masson trichrome staining was used to visualize the collagen distribution and orientation. Cell nuclei were stained using hematoxylin. Images were captured digitally using an ECLIPSE 80i microscope (Nikon, Tokyo, Japan) and qualitatively analyzed.

### Immunohistochemical (IHC) Analysis

IHC staining was performed by placing the slides (transverse and longitudinal) in an antigen retrieval solution consisting of citrate buffer at 95°C in a steamer for 10 min. After cooling, the sections were incubated in 3% perhydrol solution for 15 min to block the endogenous peroxidase reaction and non-specific binding was blocked using 1% bovine serum albumin. Sections were incubated with primary antibodies against type collagen I (Abcam, Cambridge, MA, United States, 1:200) and II (Abcam, 1:200) at 4°C overnight. Following the incubation, the slides were washed three times in PBS. Goat anti-mouse IgG1 biotinylated secondary antibody (Abcam, 1:1000) was then applied for 30 min and the slides were then subjected to three more washes in PBS followed by treatment with a streptavidin-horseradish peroxidase complex, diaminobenzidine solution, and counterstaining with haematoxylin. NM and DM samples were incubated for 5 min in 4′, 6-diamidino-2-phenylindole (DAPI, Sigma-Aldrich) for fluorescent staining of nuclei to evaluate the presence of residual nuclei.

### Analysis of DNA Content

Total genomic DNA in the samples was extracted using a Genomic DNA Extraction Kit (TaKaRa Bio, Shiga, Japan) according to the manufacturer’s instructions. Briefly, NM and DM samples were lyophilized at −80°C to thoroughly remove residual moisture until achieving a constant weight, and were then weighed, cut into thin strips and digested with proteinase K and RNase for 4 h. The digested samples were centrifuged and purified with two phenol/chloroform/isoamyl alcohol (25:24:1, v/v) 33extractions. The remaining DNA was collected using an elution buffer. The extracted genomic DNA was quantified by measuring absorbance using a NanoDrop 8000 spectrophotometer (Thermo Fisher Scientific, Waltham, MA, United States). DNA quantity was normalized to the initial dry weight of the tissue and expressed as ng/mg.

### Determination of GAG, Collagen, and Water Content

NM and DM samples were weighed, freeze-dried and weighed again. The weights before and after freeze-drying were recorded to evaluate water content. GAG content was determined using a modified dimethylmethylene blue (DMMB) method as previously described (Farndale et al., 1986). NM and DM samples were lyophilised to a constant weight and then digested at 60°C in papain buffer (125 mg/mL papain, 5 mM cysteine/HCl, 5 mM disodium EDTA in PBS) for 12 h. The absorbance values of the samples were determined at 525 nm using an Epoch Microplate Spectrophotometer (BioTek Instruments, Winooski, VT, United States) immediately after the addition of a 1,9-dimethylmethylene blue solution (Sigma-Aldrich). GAG content was calculated using a standard curve, which was made using different concentrations of chondroitin sulfate sodium salt (Sigma-Aldrich). Final values were expressed as μg of GAG per dry weight of sample. Collagen content was determined based on hydroxyproline (HYP) content, which was measured using a spectrophotometric method (Chan et al., 1998). The amount of HYP in the samples was then determined using a calibration curve prepared using HYP assay (Sigma-Aldrich), and total collagen content per mg dry weight of sample as calculated using a HYP-to-collagen ratio of 1:7.2.

### SEM

SEM was performed to examine the microarchitecture of the meniscus before and after decellularization. Meniscal samples were fixed with 2.5% (v/v) glutaraldehyde in PBS, post-fixed with 1% (w/v) OsO_4_, dehydrated using a graded alcohol series and dried using hexamethyldisilazane (Sigma-Aldrich). The dried samples were sputter-coated with gold-palladium and viewed using a model S-3000N microscope (HITACHI, Tokyo, Japan). The porosity of each group was tested by ImageJ (National Institutes of Health, United States).

### Cytotoxicity of DM Scaffold

To assess the chemical toxicity of decellularized scaffolds, cytotoxicity testing was conducted as previously described (Xu et al., 2014). Briefly, DM was incubated in standard Dulbecco’s modified Eagle medium (DMEM)/F12 containing 10% fetal bovine serum for 24 h, and the extracts was collected for subsequent use. Bone marrow mesenchymal stem cells (BMSCs) extracted from lumbar vertebra of New Zealand white rabbits were trypsinized, centrifuged and resuspended in the same medium and were maintained in culture at 37°C. And the medium was exchanged every 3 days. After successive cycles, the third-passage cells were harvested. Cells were then seeded in 96-well cell culture plates at a concentration of 2.5 × 10^4^ cells/well that would allow confluency after 24 h in standard culture medium. The medium was removed and replaced with leach liquor at varying concentrations (25, 50, and 100%). Cells cultured in the standard medium served as the control group. Cell proliferation and metabolic activity were then assessed using a Cell Counting Kit-8 (CCK-8, DOJINDO, Tokyo, Japan). CCK-8 was added to each well of the plate and incubated for 3 h on days 1, 3, 4, and 5. Absorbance was measured at 450 nm using an Epoch Microplate Spectrophotometer (BioTek, Instruments). Five replicates were evaluated per sample.

### Biomechanical Testing

All mechanical tests were performed using a model Z2.5 computer-controlled test machine (Zwick/Roell, Ulm, Germany) as we described previously (Zhao et al., 2009; Junhui et al., 2015). Mechanical uniaxial tensile tests were conducted to calculate the tensile initial elastic modulus, elastic modulus, transition stress, transition strain, and ultimate tensile strength of NM and DM. On the day of testing, frozen slices were cut from the central portion of the menisci with a cross-sectional area of 3 × 3 mm (thickness × width). In addition, to rule out the influence of damage caused by the mechanical grips, the tensile test specimens were then cut with a dumbbell-shape with a gauge length of 10 mm between the grips. Only specimens with dimensions within ± 10% of the pre-specified dimensions were considered. Specimens were kept hydrated at 37°C in PBS until testing and were then clamped to the grips in the mechanical apparatus. Tests were conducted at a strain rate of 10 mm/min, similar to our previous study (Lin et al., 2016). The initial elastic modulus and the elastic modulus were calculated from the slope of the linear curve fit up to 1% strain and the slope of the linear curve, based on the best R-square value using linear curve fitting. The intersection point of the two slopes defined the transition stress and strain. The ultimate tensile strength was calculated from the highest point on the stress strain curve.

### Recellularization *in vitro*

The cytocompatibility of the ECM scaffolds was assessed using recellularization *in vitro*. After γ-rays radiation sterilization (24KGy), the whole scaffolds were immersed in DMEM/F12 containing 10% fetal bovine serum and 1% antibiotics for 24 h and were dried using sterile gauze. New Zealand white rabbit-derived BMSCs were passaged continuously after the third generation as mentioned above. The cells were resuspended to the density of 10 × 10^6^ cells/mL. The scaffolds were then completely immersed in the cell suspension and incubated for 5 to 10 min and then transferred to a 12-well cell culture plates. Then, third-passage BMSCs were harvested and seeded onto the surface of per specimen at the same density of 2 × 10^6^ cells/cm^2^ and incubated for 2 h before the addition of supplemented culture medium. The culture medium was changed every 12 h. To generally observe the BMSCs on the scaffold on week 2 after cell seeding, cells were washed with PBS, fixed for 24 h in 10% neutral buffered formalin solution, and then stained with DAPI for 15 min at 37°C. Meanwhile, cell viability was assessed by Live-Dead cell staining, which indicated survival condition of BMSCs on scaffold after 2 weeks of culturing. The cells were washed with PBS and stained by Live-Dead cell staining kit (Biovision, Milpitas, CA, United States). After staining, cells were observed immediately using a Nikon ECLIPSE 80i microscopy (Nikon, Japan) equipped with a band-pass filter.

### *In vivo* Animal Study

The intermediate part of the meniscus was shaped into a wedge-shaped semilunar disk. Ten skeletally mature male New Zealand white rabbits weighing between 3.0 and 3.5 kg were included in this study. Partial medial meniscectomy (one-third of the meniscus) was performed on the knees of all rabbits. The right knees were transplanted with DM (DM group) and the left knees underwent operation with the NM as a control (NM group). In this procedure, the rabbits were anesthetized with intravenous urethane (1 g/kg). After skin disinfection, the right knee was approached using a medial parapatellar incision. The meniscal tissue was thawed by immersion in sterile saline solution and then sutured to the adjacent meniscus with non-resorbable No. 5-0 sutures (Ethicon, Somerville, NJ, United States) after the meniscectomy. The capsule, periarticular tissues and skin were closed with No. 3-0 sutures (Ethicon). After the operation, the animals were immediately allowed free movement without any restriction. Five of the animals were killed with pentobarbital sodium at the end of weeks 6 and 12, and samples were evaluated by histological analysis as described *above.*

### Semi-Quantification for Histological Staining

To further evaluate the repair condition of explants, semi-quantification of four typicalcells found in meniscal repair tissue was analyzed using Image J software 12 weeks H&E stained samples. Briefly, three sections of different points in both NM and DM group were chosen, respectively. The multipoint counting function in Image J was used to mark four kinds of cells, including inflammatory cells, remnant chondrocyte-like cells, fusiform cells and elongated fibroblast-like cells. Then the numbers of these cells per counting area (0.28 square millimeters) were calculated, respectively, and converted to percentage for statistical analyses.

### Statistical Analyses

All numerical data were analyzed using SPSS 19.0 software (SPSS Inc, Chicago, IL, United States). The mean and standard deviation were calculated using the Descriptive Statistics function with a 95% confidence interval. Data from NM and DM, as well as that from different test configurations were compared using two-tailed Student’s *t*-test. A *p*-value < 0.05 was considered significant.

## Results

### Histological Analysis of DM

**FIGURE 2:**
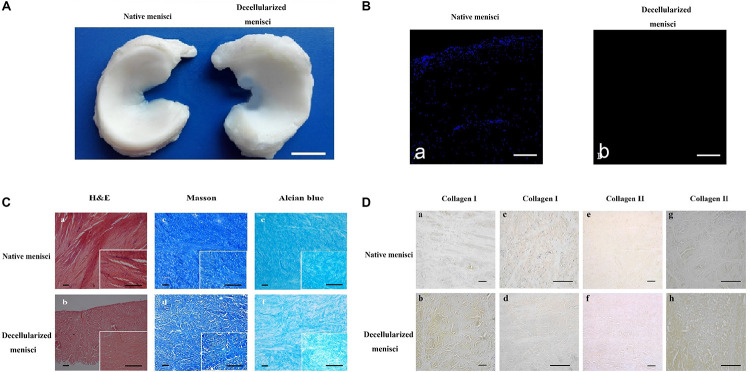
Histological and immunohistochemical analysis of native meniscus and decellularized meniscus. **(A)** Gross morphological inspections of native meniscus and meniscus scaffold. Overall shape of the scaffold was maintained after processing. Scale bar: 10 mm. **(B)** DAPI staining of longitudinal section of native and acellular meniscus scaffold **(a,b)**. Scale bar: 100 μm. **(C)** H&E staining of **(a)** native and **(b)** decellularized meniscus. Masson’s trichrome staining: **(c)** native and **(d)** decellularized meniscus; collagen is blue, cytoplasm is pink and nuclei are brown. Alcian blue staining of GAG: **(e)** native and **(f)** decellularized meniscus. Scale bar: 100 μm. **(D)** Collagen I labeling of tissue cross-sections: **(a)** native and **(b)** decellularized. Collagen I labeling of longitudinal section: **(c)** native and **(d)** decellularized. Collagen II labeling of tissue cross-sections: **(e)** native and **(f)** decellularized. Collagen II labeling of longitudinal section: **(g)** native and **(h)** decellularized. Scale bar: 100 μm.

Gross inspection (Figure 2A) revealed that the decellularization process did not change the general shape of the menisci. Histological analyses after decellularization showed complete cell removal. Compared with the NM (Figure 2Ca), the DM (Figure 2Cb) was free of cell nuclei, and the collagenous fibers were well preserved with much loose arrangement, since loosening agent was used to make decellularization easier. Masson’s trichrome staining revealed maintenance of the collagenous fibers (Figures 2Cc,d), as well as a slight increase in porosity, which is beneficial for recellularization and cell growth. Alcian blue staining (Figures 2Ce,f) revealed that GAG content was slightly reduced after decellularization, with the reduction of GAG distributed homogeneously.

### Evaluation of Collagen Organization and Expression in DM

IHC staining indicated the presence of a well-preserved collagen bundle with much loose arrangement in the DM group (Figure 2D). There was no evident change in expression of either collagen I (Figures 2Da–d) or II (Figures 2De–h) after decellularization. Strong positive staining for collagen I could be observed across the whole meniscus, while collagen II was only labeled in the fibrocartilaginous section. Besides, much stronger staining for collagen I and II could be observed in DM group compared to NM group (Figures 2Da,b,g,h). It is most likely because decellularization detergents could partially break up peptide bonds and expose more amino and carboxylic groups to form hydrogen bonding with water, which results in higher HYP and water content. A similar paradoxical phenomenon was also observed in other studies (Grauss et al., 2005; Yuan et al., 2013).

### Analysis of Residual DNA Within the Scaffold

**FIGURE 3:**
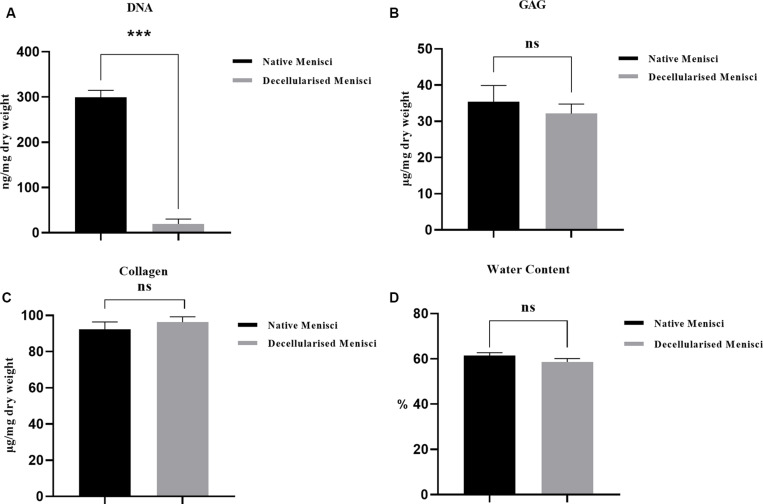
Biochemical quantification of **(A)** water (*p* < 0.05), **(B)** glycosaminoglycan (GAG) (*p* > 0.05), **(C)** hydroxyproline (HYP) (*p* > 0.05) and **(D)** DNA content (*p* < 0.001) of native and DM (*n* = 3). ***p < 0.001.

DAPI staining of nuclear content revealed an abundance of nuclei in the intact meniscus and an absence of nuclei in the scaffolds (Figure 2B). The amount of residual DNA within the DM was quantitatively evaluated using spectrophotometry, in which the peak of absorbance at 260 nm was measured. Results were normalized to the dry weight and compared with those of the NM treated with PBS (Figure 3A). The amount of DNA in NM was 299.61 ± 15.2 ng/mg dry weight, which was decreased to 19.54 ± 10.94 ng/mg dry weight (*p* < 0.001) after decellularization.

### Biochemical Analyses in DM

The DMMB assay revealed no significant difference in GAG content between NM and DM (*p* > 0.05, Supplementary Table 1 and Figure 3B). The amount of total collagen, a major component of porcine menisci, was calculated using the HYP assay. The collagen content of NM was 92.40 ± 3.98 μg/mg dry weight. There was no significant decrease in collagen content in DM (96.18 ± 3.02 μg/mg) compared with normal tissue (*p* > 0.05, Figure 3C). The percentages of water content within the menisci were 61.39 ± 1.3 vs. 58.63 ± 1.4% before and after decellularization (*p* > 0.05, Figure 3D).

### Microstructure of Scaffolds

**FIGURE 4:**
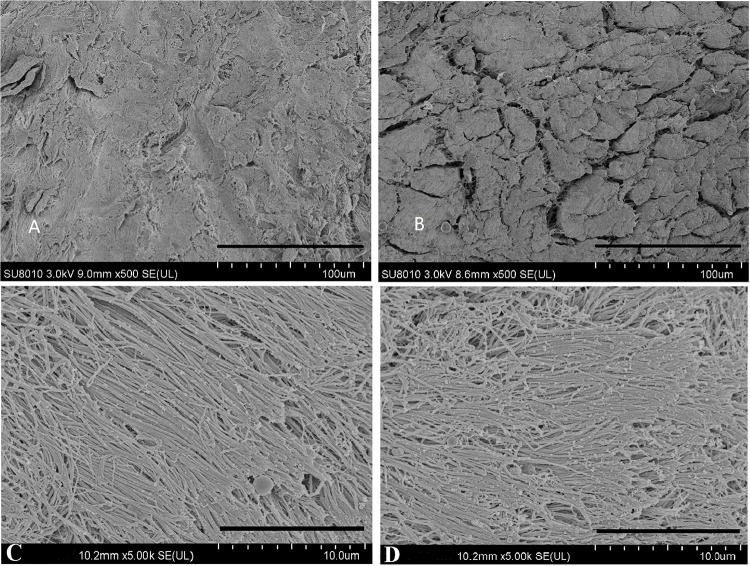
Scanning electron microscopy photographs of native menisci **(A,C)** and decellularized porcine menisci **(B,D)**. Panels **(A,B)** show the cross sections through the menisci. Scale bar: 100 μm. And the horizontal sections are demonstrated in **(C,D)**. Scale bar: 10 μm.

ECM structure was evaluated using SEM. The microstructure of NM and DM is shown in Figure 4. NM was very dense and the collagen fibers were aligned in parallel. The order of collagen fibers in the DM group was preserved, and displayed open pores after processing. The porosity in NM group and DM group was 6.5 and 19.8%, respectively.

### Cytotoxicity Testing

**FIGURE 5:**
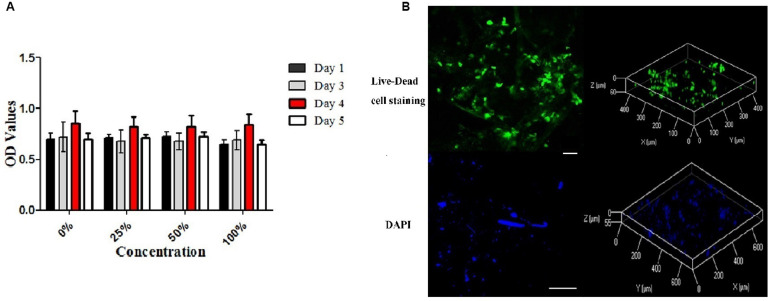
Cytotoxicity of the decellularized meniscus ECM scaffold and recellularization *in vitro*. **(A)** Cytotoxicity assay of DM: the proliferative activities of the cells cultured in standard medium and those cultured in the extracts at different concentrations. **(B)** Fluorescence Live-Dead cell (Scale bar: 30 μm) and DAPI (Scale bar: 100 μm) staining images (two and three dimensions) demonstrating seeding of BMSCs on DM ECM scaffolds at 2 weeks.

The cytotoxicity of ECM scaffolds was evaluated using a cytotoxicity assay. The metabolic activities of cells incubated in different leaching solution concentrations were assessed. CCK-8 analysis indicated that the proliferation of cells cultured in a standard medium were similar to those of cells cultured in leaching solutions (*p* > 0.05, Figure 5A).

### Biomechanical Evaluation

**FIGURE 6:**
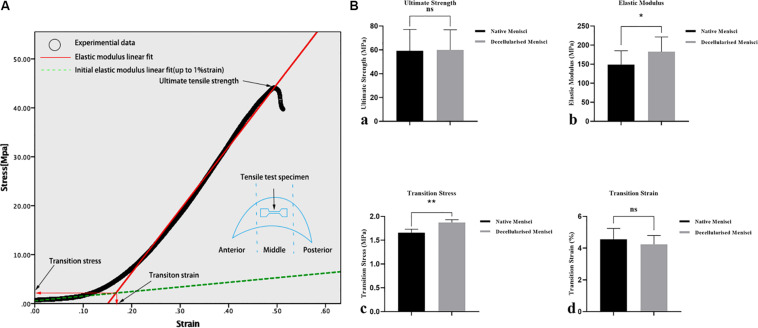
Biomechanical properties of the specimens of the native and DM (*n* = 3). **(A)** Representative curves for the tensile tests and biomechanical properties of the specimens. **(B)** Tensile mechanical properties: **(a)** ultimate strength, **(b)** transition stress, **(c)** elastic modulus and **(d)** transition strain. *p < 0.05 and **p < 0.01.

Representative curves for the results of the tensile tests are shown in Figure 6A, and the biomechanical properties of NM and DM are shown in Figure 6B. The measured initial elastic modulus, elastic modulus, ultimate tensile strength and transition stress for the NM ranged from 18.1 to 26.7, 131.0 to 167.1, 50.2 to 68.1, and 1.62 to 1.69 [MPa], respectively (Supplementary Table 2). The corresponding transition strain ranged from 3.6 to 5.8%. Compared with the NM group, the DM group showed a higher transition stress (1.66 ± 0.07 vs. 1.87 ± 0.06, p < 0.01) and elastic modulus (149.05 ± 36.25 vs. 182.70 ± 38.74, p < 0.05). The ultimate strength (59.20 ± 17.94 vs. 59.98 ± 16.93) and the transition strain (4.55 ± 0.69 vs. 4.24 ± 0.56) of DM were not significantly different from those of NM.

### Evaluation of Seeded BMSCs Integration With the ECM Scaffolds *in vitro*

After cell seeding, Live-Dead cell staining confirmed that the meniscal ECM scaffolds could support the proliferation of BMSCs *in vitro* (Figure 5B). BMSCs attached well to the surface of scaffolds and proliferated over 2 weeks, which was evident at the image of three dimensions. Also, DAPI staining demonstrated the dense distribution of BMSCs on the surface of specimens over 2 weeks. The findings further verified the cytocompatibility of the ECM scaffolds.

### *In vivo* Animal Study

**FIGURE 7:**
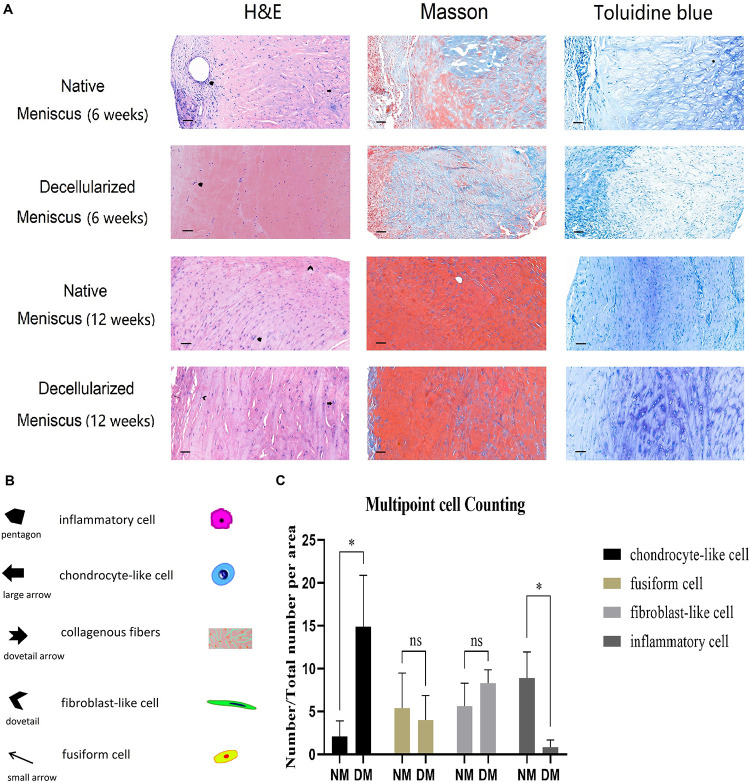
**(A)** Histological analysis (H&E, Masson’s trichrome and Toluidine Blue staining) of the native and decellularized meniscus ECM scaffolds *in vivo* after 6 weeks are showed in the 1st and 2nd row, respectively (*n* = 5). Native and decellularized meniscus ECM scaffolds after 12 weeks are in the 3nd and 4th row (*n* = 5). **(B)** The pentagon and dovetail indicate the inflammatory cell and the elongated fibroblast-like cell, respectively. The large arrow denotes the chondrocyte-like cell, and the small arrow indicates the fusiform cell. The dovetail arrow indicates collagenous fibers. Scale bar: 40 μm. **(C)** Semi-quantification of four typical cells over 12 weeks is demonstrated in the histogram (*n* = 3). The percentage of chondrocyte-like cell, fusiform cell, fibroblast-like cell and inflammatory cell are showed in column 1–4, respectively. *p < 0.05.

The scaffolds were harvested after 6 and 12 weeks, respectively. Histological analysis (H&E, Masson’s trichrome and Toluidine Blue staining) of the NM group after 6 weeks demonstrated the appearance of infiltrates of inflammatory cells, remnant chondrocyte-like cells and elongated fibroblast-like cells in clumps and collagenous fibers arranged in order. In contrast, the DM group 6 weeks after surgery displayed few chondrocyte-like cells and fibroblast-like cells with infiltrates of inflammatory cells (Figure 7). After 12 weeks, fibrocartilage differentiation was detected with cells of three phenotypes surrounded by ECM in the DM group, while poor differentiation was observed for the NM group (Figure 7).

### Semi-Quantification for Histological Staining

After 12 weeks, three sections of different points in both NM and DM group were used for semi-quantification of H&E analysis. Cell numbers were calculated and converted to percentage of total cells (Figure 7). There was no statistical difference for fusiform cells and fibroblast-like cells. However, there were more chondrocyte-like cells in the DM group than NM group (*p* < 0.05). Additionally, there were more inflammatory cells in the NM group than DM group (*p* < 0.05). The results showed the better repair ability and milder inflammatory reaction of the scaffolds *in vivo.*

## Discussion

In this study, a new protocol was developed for the decellularization of whole porcine meniscus. It was confirmed that BMSCs attached well to the surface of scaffolds and proliferated over 2 weeks of culture on the decellularized meniscus scaffold *in vitro*. Additionally, after 12 weeks, region-specific recellularization was detected in the DM group *in vivo*, while poor differentiation for the NM group. Finally, better repair ability and milder inflammatory reaction of the scaffolds were confirmed using histological semi-quantification.

To date, menisci from a variety of species have been decellularized (Maier et al., 2007; Stapleton et al., 2008; Sandmann et al., 2009; Stabile et al., 2010; Schwarz et al., 2012; Azhim et al., 2013; Chen et al., 2015; Wu et al., 2015). However, entire meniscal ECM scaffolds were developed in only a few studies (Maier et al., 2007; Sandmann et al., 2009; Stabile et al., 2010). Also, considering the properties of these scaffolds for whole meniscal replacement, no optimal scaffold that could maintain tissue ECM and preserve biomechanical properties together with minimum DNA residues in parallel has been developed. In 2008, Sandmann et al. (2009) decellularized human menisci with 2% SDS for 2 weeks, and complete removal of cells was achieved without compromising biomechanical properties. However, there was no evaluation about the *in vivo* performance of the scaffolds. It was confirmed that SDS was more efficient in cell lysis than Triton X-100, but the former was observed to cause extreme fragmentation and swelling of collagen fibers (Andrew, 1986; Courtman et al., 1994). Also, SDS separates GAGs from proteins in the ECM and is less supportive of cell reseeding of the scaffolds (Maier et al., 2007). Therefore, the time of SDS treatment should be reduced to ensure GAG retention.

In this study, two kinds of chemical detergents (SDS and Triton X-100) were used for decellularization. We used different detergent concentrations and processing time and determined an optimal protocol, which effectively removed all the macroscopic nuclei and preserved the ECM. In this protocol, non-ionic detergents such as Triton X-100 are more effective than ionic detergents such as SDS. Therefore, utilizing Triton X-100 as decellularization reagent first is more conducive to the subsequent decellularization process. SDS appears more effective than Triton X-100 for removing nuclei. Such an optimal protocol can make the difference between complete and incomplete cell residues and nuclei removal. To minimize the DNA content in the ECM, DNAase I was applied to decompose the residual nuclear fragments in the ECM after the detergent processing.

The DNA content within ECM scaffolds decreased by more than 90% and was reduced to an acceptable level according to minimal criteria which suffice to satisfy the intent of decellularization (<50 ng/mg dry weight) (Crapo et al., 2011). H&E and DAPI staining confirmed the relatively successful removal of cell nuclei. Immunohistochemical staining indicated the preservation of collagen I and II. In addition, biochemical assays showed a favorable maintenance of water, GAG and collagen content. SEM was conducted to evaluate the microstructure. The main microstructure characteristics were well preserved while porosity of the menisci was increased, which may be beneficial for cell attachment and proliferation. Cytotoxicity assays demonstrated similar growth trend of cells in a standard medium and leaching solution of DM, which confirmed the absence of chemical residue within DM after decellularization.

As for the biomechanical properties, the tensile biomechanical properties of DM were compared with NM. The elastic modulus of DM was higher than that of NM, which was similar to that of a previous study (Abdelgaied et al., 2015), mainly because of the loss of tissue components within the accepted range. The ultimate strength and the transition strain of DM were not significantly different from those of NM. These results demonstrated that the biomechanical properties of porcine menisci were successfully maintained during decellularization. In previous studies, GAG extraction changed the structure of the ECM, increasing compressive stiffness and compressibility and slightly decreasing the residual force (Maier et al., 2007; Azhim et al., 2013). The extraction of GAGs resulted in the loss of water and thus contributing to the increase of stiffness (Chen et al., 2017). Thus, the comprehensive analyses performed to evaluate this scaffold were more extensive than those in previous studies.

The biomechanical property of the meniscus is deeply influenced by its anisotropic architecture. The meniscus is characterized by regional differences in composition, structure and cell phenotype. Generally, the outer region of the meniscus is characterized by highly aligned collagen type I fibers and is full of elongated fibroblast-like cells. Whereas the inner region becomes less aligned and has more GAGs than the outer zone, which has much higher GAG content and rounded chondrocyte-like cells and is dominated by collagen type II network (Makris et al., 2011). The microarchitecture plays an important role in regulating the behaviors of endogenous or exogenous stem/progenitor cells and subsequent tissue formation (Kumar et al., 2011; Neffe et al., 2015). Efforts have been made in recent studies to mimic the organization and interactions among the major tissue constituents of natural meniscus. In this study, region-specific extracellular matrix was preserved well after decellularization, providing biomechanical properties that were more similar to the native porcine meniscus than that of the other acellular scaffolds previously reported (Stapleton et al., 2008; Chen et al., 2015). In addition, both *in vitro* and *in vivo* studies have confirmed that the mean pore size of scaffold would also influence fibrochondrogenic differentiation and subsequent tissue formation (Makris et al., 2011). Larger pores of scaffolds benefit cell diffusion and migration, while smaller ones provide a higher surface area for cell adhesion (O’Brien et al., 2005). An anisotropic architecture of these pores serves as templates to guide extracellular matrix deposition to mimic its native counterpart, which may contribute to the biochemical and mechanical properties of regenerated constructs. In this study, the porosity of meniscus was promoted significantly after decellularization. Excellent region-specific recellularization was achieved *in vivo*.

## Conclusion

We prepared a novel porcine meniscal ECM scaffold that preserved bioactivity, biomechanics and cytocompatibility features, and promoted excellent region-specific recellularization. In conclusion, the acellular xenogeneic meniscal scaffold has excellent potential for development of a tissue-engineered solution for meniscal repair.

## Data Availability Statement

All datasets generated for this study are included in the article/Supplementary Material.

## Ethics Statement

The animal study was reviewed and approved by the Animal Experimental Ethics Committee of Zhejiang University.

## Author Contributions

All authors listed have made a substantial, direct and intellectual contribution to the work, and approved it for publication.

## Conflict of Interest

The authors declare that the research was conducted in the absence of any commercial or financial relationships that could be construed as a potential conflict of interest.

## References

[B1] Abdelgaied A. Stanley M. Galfe M. Berry H. Ingham E. Fisher J. (2015). Comparison of the biomechanical tensile and compressive properties of decellularised and natural porcine meniscus. *J. Biomech.* 48 1389–1396. 10.1016/j.jbiomech.2015.02.044 25766391

[B2] Andrew E. (1986). Damage of porcine aortic valve tissue caused by the surfactant sodiumdodecylsulphate. *Thorac. Cardiovasc. Surg.* 34 340–341. 10.1055/s-2007-1020381 2431511

[B3] Azhim A. Ono T. Fukui Y. Morimoto Y. Furukawa K. Ushida T. (2013). Preparation of decellularized meniscal scaffolds using sonication treatment for tissue engineering. *Conf. Proc. IEEE. Eng. Med. Biol. Soc.* 2013 6953–6956. 10.1109/EMBC.2013.6611157 24111344

[B4] Bruns J. Kahrs J. Kampen J. Behrens P. Plitz W. (1998). Autologous perichondral tissue for meniscal replacement. *J. Bone Joint Surg. Br.* 80 918–923. 10.1302/0301-620x.80b5.8023 9768910

[B5] Cameron J. C. Saha S. (1997). Meniscal allograft transplantation for unicompartmental arthritis of the knee. *Clin. Orthop. Relat. Res.* 337 164–171. 10.1097/00003086-199704000-199704018 9137187

[B6] Cartmell J. S. Dunn M. G. (2004). Development of cell-seeded patellar tendon allografts for anterior cruciate ligament reconstruction. *Tissue Eng.* 10 1065–1075. 10.1089/ten.2004.10.1065 15363164

[B7] Chan B. P. Fu S. C. Qin L. Rolf C. Chan K. M. (1998). Pyridinoline in relation to ultimate stress of the patellar tendon during healing: an animal study. *J. Orthop. Res.* 16 597–603. 10.1002/jor.1100160512 9820284

[B8] Chen Y. Chen J. Zhang Z. Lou K. Zhang Q. Wang S. (2017). Current advances in the development of natural meniscus scaffolds: innovative approaches to decellularization and recellularization. *Cell Tissue Res.* 370 41–52. 10.1007/s00441-017-2605-0 28364144 PMC5610206

[B9] Chen Y. C. Chen R. N. Jhan H. J. Liu D. Z. Ho H. O. Mao Y. (2015). Development and characterization of acellular extracellular matrix scaffolds from porcine menisci for use in cartilage tissue engineering. *Tissue Eng Part C Methods* 21 971–986. 10.1089/ten.TEC.2015.0036 25919905 PMC4553380

[B10] Cole B. J. Dennis M. G. Lee S. J. Nho S. J. Kalsi R. S. Hayden J. K. (2006). Prospective evaluation of allograft meniscus transplantation: a minimum 2-year follow-up. *Am. J. Sports Med.* 34 919–927. 10.1177/0363546505284235 16476914

[B11] Courtman D. W. Pereira C. A. Kashef V. McComb D. Lee J. M. Wilson G. J. (1994). Development of a pericardial acellular matrix biomaterial: biochemical and mechanical effects of cell extraction. *J. Biomed. Mater. Res.* 28 655–666. 10.1002/jbm.820280602 8071376

[B12] Crapo P. M. Gilbert T. W. Badylak S. F. (2011). An overview of tissue and whole organ decellularization processes. *Biomaterials* 32 3233–3243. 10.1016/j.biomaterials.2011.01.057 21296410 PMC3084613

[B13] Ding C. Martel-Pelletier J. Pelletier J. P. Abram F. Raynauld J. P. Cicuttini F. (2007). Meniscal tear as an osteoarthritis risk factor in a largely non-osteoarthritic cohort: a cross-sectional study. *J. Rheumatol.* 34 776–784. 10.1097/01.rhu.0000260650.43402.b6 17361984

[B14] Farndale R. W. Buttle D. J. Barrett A. J. (1986). Improved quantitation and discrimination of sulphated glycosaminoglycans by use of dimethylmethylene blue. *Biochim. Biophys. Acta* 883 173–177. 10.1016/0304-4165(86)90306-90305 3091074

[B15] Gilbert T. W. Sellaro T. L. Badylak S. F. (2006). Decellularization of tissues and organs. *Biomaterials* 27 3675–3683. 10.1016/j.biomaterials.2006.02.014 16519932

[B16] Grauss R. W. Hazekamp M. G. Oppenhuizen F. van Munsteren C. J. Gittenberger-de Groot A. C. DeRuiter M. C. (2005). Histological evaluation of decellularised porcine aortic valves: matrix changes due to different decellularisation methods. *Eur. J. Cardiothorac. Surg.* 27 566–571. 10.1016/j.ejcts.2004.12.052 15784352

[B17] Ha J. K. Sung J. H. Shim J. C. Seo J. G. Kim J. G. (2011). Medial meniscus allograft transplantation using a modified bone plug technique: clinical, radiologic, and arthroscopic results. *Arthroscopy* 27 944–950. 10.1016/j.arthro.2011.02.013 21693347

[B18] Ionescu L. C. Lee G. C. Garcia G. H. Zachry T. L. Shah R. P. Sennett B. J. (2011). Maturation state-dependent alterations in meniscus integration: implications for scaffold design and tissue engineering. *Tissue Eng Part A* 17 193–204. 10.1089/ten.TEA.2010.0272 20712419 PMC3011923

[B19] Junhui L. Zhengfeng M. Zhi S. Mamuti M. Lu H. Shunwu F. (2015). Anchorage of annulus fibrosus within the vertebral endplate with reference to disc herniation. *Microsc. Res. Tech.* 78 754–760. 10.1002/jemt.22536 26178646

[B20] Klompmaker J. Veth R. P. Jansen H. W. Nielsen H. K. de Groot J. H. Pennings A. J. (1996). Meniscal replacement using a porous polymer prosthesis: a preliminary study in the dog. *Biomaterials* 17 1169–1175. 10.1016/0142-9612(96)84937-84934 8799501

[B21] Kohn D. Rudert M. Wirth C. J. Plitz W. Reiss G. Maschek H. (1997). Medial meniscus replacement by a fat pad autograft. an experimental study in sheep. *Int. Orthop.* 21 232–238. 10.1007/s002640050157 9349959 PMC3617697

[B22] Kumar G. Tison C. K. Chatterjee K. Pine P. S. McDaniel J. H. Salit M. L. (2011). The determination of stem cell fate by 3D scaffold structures through the control of cell shape. *Biomaterials* 32 9188–9196. 10.1016/j.biomaterials.2011.08.054 21890197 PMC3428125

[B23] Lin X. Fang X. Wang Q. Hu Z. Chen K. Shan Z. (2016). Decellularized allogeneic intervertebral disc: natural biomaterials for regenerating disc degeneration. *Oncotarget* 7 12121–12136. 10.18632/oncotarget.7735 26933821 PMC4914273

[B24] Maier D. Braeun K. Steinhauser E. Ueblacker P. Oberst M. Kreuz P. C. (2007). In vitro analysis of an allogenic scaffold for tissue-engineered meniscus replacement. *J. Orthop. Res.* 25 1598–1608. 10.1002/jor.20405 17676613

[B25] Makris E. A. Hadidi P. Athanasiou K. A. (2011). The knee meniscus: structure–function, pathophysiology, current repair techniques, and prospects for regeneration. *Biomaterials* 32 7411–7431. 10.1016/j.biomaterials.2011.06.037 21764438 PMC3161498

[B26] Messner K. Kohn D. Verdonk R. (1999). Future research in meniscal replacement. *Scand. J. Med. Sci. Sports* 9 181–183. 10.1111/j.1600-0838.1999.tb00451.x 10380277

[B27] Milachowski K. A. Weismeier K. Wirth C. J. (1989). Homologous meniscus transplantation. *Exp. Clin. Results Int. Orthop.* 13 1–11. 10.1007/bf00266715 2722311

[B28] Neffe A. T. Pierce B. F. Tronci G. Ma N. Pittermann E. Gebauer T. (2015). One step creation of multifunctional 3D architectured hydrogels inducing bone regeneration. *Adv. Mater. Weinheim* 27 1738–1744. 10.1002/adma.201404787 25601165

[B29] Noyes F. R. Barber-Westin S. D. (2010). Repair of complex and avascular meniscal tears and meniscal transplantation. *J. Bone Joint Surg. Am.* 92 1012–1029. 20360529

[B30] Noyes F. R. Barber-Westin S. D. Rankin M. (2005). Meniscal transplantation in symptomatic patients less than fifty years old. *J. Bone Joint Surg. Am.* 87(Suppl. 1), 149–165. 10.2106/JBJS.E.00347 16140791

[B31] O’Brien F. J. Harley B. A. Yannas I. V. Gibson L. J. (2005). The effect of pore size on cell adhesion in collagen-GAG scaffolds. *Biomaterials* 26 433–441. 10.1016/j.biomaterials.2004.02.052 15275817

[B32] Rath E. Richmond J. C. Yassir W. Albright J. D. Gundogan F. (2001). Meniscal allograft transplantation. Two- to eight-year results. *Am. J. Sports Med.* 29 410–414. 10.1177/03635465010290040401 11476377

[B33] Sandmann G. H. Eichhorn S. Vogt S. Adamczyk C. Aryee S. Hoberg M. (2009). Generation and characterization of a human acellular meniscus scaffold for tissue engineering. *J. Biomed. Mater. Res. A* 91 567–574. 10.1002/jbm.a.32269 18985757

[B34] Schwarz S. Koerber L. Elsaesser A. F. Goldberg-Bockhorn E. Seitz A. M. Dürselen L. (2012). Decellularized cartilage matrix as a novel biomatrix for cartilage tissue-engineering applications. *Tissue Eng Part A* 18 2195–2209. 10.1089/ten.TEA.2011.0705 22690787

[B35] Stabile K. J. Odom D. Smith T. L. Northam C. Whitlock P. W. Smith B. P. (2010). An acellular, allograft-derived meniscus scaffold in an ovine model. *Arthroscopy* 26 936–948. 10.1016/j.arthro.2009.11.024 20620793

[B36] Stapleton T. W. Ingram J. Fisher J. Ingham E. (2011). Investigation of the regenerative capacity of an acellular porcine medial meniscus for tissue engineering applications. *Tissue Eng Part A* 17 231–242. 10.1089/ten.TEA.2009.0807 20695759 PMC3011925

[B37] Stapleton T. W. Ingram J. Katta J. Knight R. Korossis S. Fisher J. (2008). Development and characterization of an acellular porcine medial meniscus for use in tissue engineering. *Tissue Eng Part A* 14 505–518. 10.1089/tea.2007.0233 18370607

[B38] Stone K. R. Rodkey W. G. Webber R. McKinney L. Steadman J. R. (1992). Meniscal regeneration with copolymeric collagen scaffolds. in vitro and in vivo studies evaluated clinically, histologically, and biochemically. *Am. J. Sports Med.* 20 104–111. 10.1177/036354659202000202 1558234

[B39] van Arkel E. R. de Boer H. H. (2002). Survival analysis of human meniscal transplantations. *J. Bone Joint Surg. Br.* 84 227–231. 10.1302/0301-620x.84b2.12443 11924652

[B40] Verdonk R. (2002). Meniscal transplantation. *Acta Orthop. Belg.* 68 118–127. 10.1097/01.BTK.0000023922.35146.4EV 12050996

[B41] Wu J. Ding Q. Dutta A. Wang Y. Huang Y. H. Weng H. (2015). An injectable extracellular matrix derived hydrogel for meniscus repair and regeneration. *Acta Biomater.* 16 49–59. 10.1016/j.actbio.2015.01.027 25644450

[B42] Xu H. Xu B. Yang Q. Li X. Ma X. Xia Q. (2014). Comparison of decellularization protocols for preparing a decellularized porcine annulus fibrosus scaffold. *PLoS one* 9:e86723. 10.1371/journal.pone.0086723 24475172 PMC3901704

[B43] Yuan M. Yeung C. W. Li Y. Y. Diao H. Cheung K. Chan D. (2013). Effects of nucleus pulposus cell-derived acellular matrix on the differentiation of mesenchymal stem cells. *Biomaterials* 34 3948–3961. 10.1016/j.biomaterials.2013.02.004 23465833

[B44] Zhao F. D. Pollintine P. Hole B. D. Adams M. A. Dolan P. (2009). Vertebral fractures usually affect the cranial endplate because it is thinner and supported by less-dense trabecular bone. *Bone* 44 372–379. 10.1016/j.bone.2008.10.048 19049912

