# Supplementary material for: Preparation and Characterization of an Optimized Meniscal Extracellular Matrix Scaffold for Meniscus Transplantation
Source: Front Bioeng Biotechnol. 2020 Jul 9;8:779. doi: 10.3389/fbioe.2020.00779 (PMC7381338; doi:10.3389/fbioe.2020.00779)
Supplement: Supplementary file 1 [file Table_1.doc]

**Table. 1.** Water, GAG, collagen and DNA content of normal and decellularized menisci.

| Component | Normal meniscal tissue | Decellularized meniscal tissue | *P* value |
| --- | --- | --- | --- |
| Water  GAG  Collagen  DNA | 61.39 ± 1.3%  35.39 ± 4.49 μg/mg dry weight  92.40 ± 3.98 μg/mg dry weight  299.61 ± 15.2 ng/mg dry weight | 58.63 ± 1.4%  32.15 ± 2.64 μg/mg dry weight  96.18 ± 3.02 μg/mg dry weight  19.54 ± 10.94 ng/mg dry weight | *p* = 0.073  *p* = 0.188  *p* = 0.806  ****p* < 0.001 |
