# Supplementary material for: Preparation and Characterization of an Optimized Meniscal Extracellular Matrix Scaffold for Meniscus Transplantation
Source: Front Bioeng Biotechnol. 2020 Jul 9;8:779. doi: 10.3389/fbioe.2020.00779 (PMC7381338; doi:10.3389/fbioe.2020.00779)
Supplement: Supplementary file 2 [file Table_2.doc]

**Table. 2. Tensile mechanical properties for NM and DM (3.0 × 3.0 × 10 [mm] samples, mean ± 95% CI) at a strain rate of 10 mm/min.**

| Parameter | Normal meniscal tissue | Decellularized meniscal tissue | *P* value |
| --- | --- | --- | --- |
| Initial elastic modulus Transition stress Elastic modulus Ultimate strength  Transition strain | 22.43 ± 8.62  1.66 ± 0.07  149.05 ± 36.25  59.20 ± 17.94  4.55 ± 0.69 | 19.07 ± 6.96  1.87 ± 0.06  182.70 ± 38.74  59.98 ± 16.93  4.24 ± 0.56 | *p* = 0.207  ***p* < 0.01  * *p* < 0.05  *p* = 0.895  *p* = 0.15 |
